# Biomaterials for bone infections: antibacterial mechanisms and immunomodulatory effects

**DOI:** 10.3389/fcimb.2025.1589653

**Published:** 2025-05-27

**Authors:** Haoran Wang, Hongxia Li, Shengnan Jin, Fang Yan, Xiaoyu Qu, Xi Chen, Zishan Peng, Linping Wang, Junying Li, Yang Liu

**Affiliations:** ^1^ College of Integrated Traditional Chinese and Western Medicine, Liaoning University of Traditional Chinese Medicine, Shenyang, Liaoning, China; ^2^ Department of Laboratory Medicine, Shengjing Hospital of China Medical University, Shenyang, Liaoning, China; ^3^ Innovative Engineering Technology Research Center for Cell Therapy, Shengjing Hospital of China Medical University, Shenyang, Liaoning, China; ^4^ Department of Gastroenterology, Shengjing Hospital of China Medical University, Shenyang, Liaoning, China

**Keywords:** bone infection, immune cells, biomaterials, scaffolds, macrophages

## Abstract

As the global population continues to age, an increasing number of individuals suffer from osteoporosis, fractures, bone infections, and bone tumors. Among these, bone infection is considered one of the most challenging clinical infections due to its high recurrence rate, bacterial resistance, high incidence, and substantial treatment costs. However, these challenges underscore the urgent need for clinicians to develop novel therapeutic strategies to improve the current cure rate and reduce the mortality associated with bone infections. Current scientific research on bone infections primarily focuses on developing new antibacterial targets and infection-resistant biomaterials. In recent years, remarkable advancements have been made in anti-infective biomaterials, offering promising solutions to overcome bone infections. By optimizing the biological properties of biomaterials or integrating them with other materials, researchers aim to achieve maximum antibacterial efficacy and biocompatibility. Such advancements enhance the integration of biomaterials with soft tissues, improve interactions between bone cells and biomaterials, promote osteogenesis, and mitigate inflammatory responses. This review primarily focuses on exploring the antibacterial mechanisms of infection-resistant biomaterials and their regulatory effects on the immune system, with particular emphasis on nanoscale carriers, scaffolds, and particulate materials.

## 1 Introduction

In joint replacement surgery, the hip and knee joints are the most frequently replaced each year. Due to their unique anatomical characteristics, including poor blood supply and high mobility, these joints are particularly susceptible to postoperative infections and bone wear. Among the most challenging and complex clinical issues faced by orthopedic surgeons is postoperative infection associated with orthopedic material implantation (Vallet-Regí et al., 2020). Studies of total knee replacement in Medicare beneficiaries show that postoperative infection is one of the important causes of surgical failure and long-term disability in patients. In addition, seasonal factors may also influence the rate of infection after total hip replacement. During material packaging or prosthesis implantation, bacteria and other microorganisms may adhere to the biomaterial surface, subsequently leading to infection, as the biomaterial surface provides an ideal substrate for pathogen adhesion (Maradit Kremers et al., 2015). A population-based study has shown that between 1969 and 2009, the incidence of osteomyelitis varied in different age groups and populations, but the overall trend showed a certain increase. This is closely related to an aging population, an increase in chronic diseases, and advances in medical technology, such as the widespread practice of orthopedic surgery (Qin et al., 2024). Additionally, a study has found that the incidence of fracture-related infections varies among different regions and populations, and the emergence of antibiotic-resistant strains has increased the complexity of treatment (Zhang et al., 2022). Variations in surgical skill levels among clinicians result in differences in prosthesis-bone congruence. Persistent micromotion of the prosthesis can cause surrounding bone wear or even fragmentation, leading to inflammation characterized by redness, swelling, heat, and pain. Prolonged wear may also result in necrosis, providing an optimal site for bacterial colonization. Despite continuous advancements in biomaterial performance and improvements in sterile surgical techniques, the infection rate and mortality associated with prosthetic implantation have significantly decreased; however, infections still occur (Saeed et al., 2018). The primary pathogens responsible for these infections are Staphylococcus aureus and Escherichia coli, with Staphylococcus aureus being the predominant causative agent of chronic osteomyelitis and bone abscesses, typically transmitted via hematogenous spread or traumatic infection. The widespread overuse of antibiotics in clinical practice has led to the development of antibiotic resistance in many pathogens, particularly Staphylococcus aureus, significantly diminishing the efficacy of antibiotics (Arciola et al., 2005). Staphylococcus aureus employs various strategies to evade immune cell-mediated destruction, including the direct release of virulence factors that suppress immune cell function or the formation of biofilms that prevent immune cell recognition and antigen presentation (Hall-Stoodley et al., 2004). Consequently, altering drug delivery methods and targeting mechanisms to enhance the antibacterial efficacy of antibiotics has become a key focus in drug development. Strategies such as nano scaffolds and vesicle-based drug delivery are being explored.

With the increasing clinical demand for implantable materials in recent years, extensive research has been conducted on bone-related tissue engineering, leading to the development of numerous anti-infective biomaterials that have demonstrated significant improvements in antibacterial properties and bone regeneration (Nair et al., 2011). However, the immune response of immune cells to biomaterials remains poorly understood. Implant rejection induces the release of cytokines and other substances by damaged cells, attracting immune cells to aggregate and infiltrate the infection site, triggering an inflammatory response (Nieminen et al., 2008). The excessive release of inflammatory cytokines and chemokines by immune cells promotes the differentiation of bone cells into osteoclasts and induces macrophages to differentiate into osteoclasts, thereby disrupting the balance between osteoclasts and osteoblasts, enhancing bone resorption while inhibiting bone formation, and ultimately leading to bone destruction. Furthermore, if immune cells and antibiotics fail to eliminate pathogens effectively, acute infections may progress into chronic infections, further hindering bone healing and biomaterial integration. Chronic inflammation leads to granulation tissue formation, bone wear, and immune cell accumulation around the affected bone and soft tissues, significantly increasing the difficulty of treatment (Sadtler et al., 2019).

Among immune cells, macrophages exhibit the highest plasticity. Some biomaterials regulate immune responses at the infection site by modulating macrophage states. For instance, one study demonstrated that modifying the surface roughness and hydrophilicity of titanium-activated macrophages polarizes them into an anti-inflammatory phenotype, thereby reducing inflammatory responses *in vivo* (Abaricia et al., 2020a). Additionally, the chemical composition and wettability of biomaterial surfaces influence cell activity and responsiveness. By altering the hydrophilicity and roughness of titanium alloy surfaces, macrophage polarization can be regulated; rough titanium alloy surfaces promote macrophage polarization toward the M2 anti-inflammatory phenotype, increasing IL-4 and IL-10 expression levels. These modifications improve immune responses and promote wound healing (Hotchkiss et al., 2016). During inflammatory responses, neutrophils are the first and most abundant immune cells to reach the infection site. They clear infected tissues and cellular debris while secreting chemokines that recruit other immune cells, including macrophages and natural killer (NK) cells (Blum et al., 2021). However, excessive neutrophil activation can damage surrounding bone and soft tissues through the release of matrix metalloproteinases and peroxidases (Chang et al., 2003). Thus, modifying biomaterial surface properties to regulate neutrophil activation is a promising strategy. Studies have shown that smooth titanium alloy surfaces exhibit significantly increased peroxidase and metalloproteinase levels, though the underlying mechanisms remain unclear (Abaricia et al., 2020b). T cells are classified into helper T cells and cytotoxic T cells, with helper T cells differentiating into various subtypes, including Th1, Th2, and Th17, depending on cytokine stimulation in the microenvironment (Adusei et al., 2021). Both Th1 and Th2 cells activate macrophages, with Th2 cells also inducing immune responses against parasites and contributing to allergic reactions (Gordon, 2003). Th17 cells promote immune cell aggregation and cytokine production by releasing Th17A (Iwakura et al., 2011).

Clinically, prosthetic infections are typically managed with antibiotic therapy. In cases of severe infection or significant prosthetic loosening, surgical removal of the prosthesis and thorough debridement may be necessary. While this approach effectively resolves infections, it imposes considerable financial and physical burdens on patients. Additionally, latent bacteria are difficult to eradicate, contributing to the development of chronic inflammation. From a preventive and therapeutic perspective, not only is the development of novel antibiotics essential, but enhancing the antibacterial properties and biocompatibility of biomaterials is also critical to achieving optimal infection control. This review focuses on elucidating the antibacterial mechanisms of infection-resistant biomaterials and their immunomodulatory effects, particularly those associated with nanocarriers, scaffolds, and particulate materials.

## 2 Silver nanomaterials

Nanomaterials have experienced substantial growth in tissue engineering, with silver nanomaterials emerging as a research hotspot due to their unique physical and biological properties. Silver exhibits excellent antibacterial activity and biocompatibility, making it a promising candidate for applications in diagnostics and anticancer therapy (Ninan et al., 2020). Researchers continue to explore superior antibacterial materials, with the antimicrobial properties of silver nanomaterials documented since early studies, leading to their incorporation into medical materials and disinfectants. Concurrently, scientists are investigating the specific antibacterial mechanisms of silver nanomaterials, particularly their interactions with immune cells. Osteomyelitis pathogens invade the bone marrow via the bloodstream or wounds, causing severe tissue destruction, particularly in bone and surrounding soft tissues. Effective infection control is crucial for successful treatment (Inzana et al., 2016). Chronic osteomyelitis is characterized by extensive bone destruction (sequestrum formation), necessitating surgical debridement and implant placement. However, implant surgery carries an inherent infection risk. Studies have demonstrated that silver ions enhance osteomyelitis treatment efficacy, particularly in terms of antibacterial activity. Lu et al. incorporated silver ions and titanium dioxide into porous nano-hydroxyapatite/polyamide 66 scaffolds to enhance their antibacterial performance and biocompatibility. In a rabbit osteomyelitis model, the modified material exhibited superior antibacterial efficacy and therapeutic outcomes, promoting osteoblast adhesion and proliferation while accelerating bone formation at the osteomyelitis site. Importantly, the material displayed excellent biocompatibility with no detectable toxicity *in vivo* (Lu et al., 2016). The surface of implants serves as an optimal niche for pathogen colonization. Modifying implant surface physicochemical properties enhances antibacterial properties. Diamond-like carbon (DLC) coatings containing silver nanoparticles exhibit superior resistance against Staphylococcus aureus and Staphylococcus epidermidis compared to non-silver-coated specimens. DLC releases silver ions that eliminate pathogens while promoting endothelial cell adhesion within 24 hours, facilitating wound healing by forming a confluent cell layer within seven days (Gorzelanny et al., 2016). In a mouse model of chronic osteomyelitis infected with Staphylococcus aureus, silver ion-containing hydroxyapatite films effectively prevented implant infections. Despite a transient increase in IL-6 during acute infection, IL-6 and C-reactive protein levels were significantly lower in the silver-treated group during later stages, indicating that silver ions suppress Staphylococcus aureus infections and enhance immune responses (Funao et al., 2016). Further, incorporating growth factors and silver into hydroxyapatite coatings enhances both antibacterial and osteogenic properties. These coatings effectively inhibit Staphylococcus aureus and Escherichia coli proliferation while promoting bone marrow stromal cell differentiation and new bone formation in rabbit femur models (Xie et al., 2014). Additionally, silver ions modulate immune cell responses. You et al. developed silver nanoparticle-collagen/chitosan scaffolds (Nag-CCS) for full-thickness skin defects in rats, demonstrating reduced pro-inflammatory and scar-related factors, upregulated M2 macrophage markers, and decreased IL-6 and TNF-α levels, facilitating wound healing and exhibiting superior antibacterial performance (You et al., 2017).

## 3 Titanium alloy composite biomaterials

With the advent of an aging population, the demand for titanium alloy implants is increasing. However, implant-associated infections remain one of the most severe postoperative complications, leading to prolonged hospital stays and increased treatment costs (Carey et al., 2020). The infection risk of titanium alloy implants has been rising annually, posing a significant challenge in orthopedic surgeries. To mitigate this risk and accelerate fracture healing, researchers have developed antibacterial coatings for titanium alloy surfaces (Shao et al., 2022). However, most antibacterial coatings can only eliminate invading pathogens for a limited time and do not assist immune cells in establishing immune memory. B lymphocytes are unable to effectively release antibodies to counter future pathogen invasions, and these coatings are often difficult to degrade, impeding bone healing. Lian et al. developed a three-layer sandwich-structured titanium alloy coating, in which vancomycin constitutes the outer and inner layers, while sodium alginate containing IL-12 liposomes forms the middle layer. This coating structure not only prolongs antimicrobial activity but also allows IL-12 to regulate the local immune microenvironment by activating immune cells for pathogen clearance and establishing immune memory. IL-12 promotes Th1 cell differentiation, activates macrophages, and stimulates B lymphocytes to produce antibodies (Carey et al., 2020). However, titanium particles can sometimes trigger implant failure due to immune system abnormalities. Titanium dental implants release titanium particles, and transcriptomic analysis of gingival tissues from non-implanted, failed, and successful implant cases revealed a significant presence of lymphocytes and macrophages in failed implants. The expression levels of IL-1β, IL-8, and IL-18 were significantly elevated, indicating that titanium particles disrupt immune microenvironment balance by modulating lymphocyte and macrophage polarization, leading to osseointegration failure (Kheder et al., 2023). In another study, it was found that debridement altered the microstructure of the titanium surface, inducing macrophage and CD4+ T cell inflammatory differentiation. The percentage of M1 macrophages and Th17 cells, along with the expression of inflammatory factors, increased. However, incorporating the glycolysis inhibitor 2-DG on the titanium surface suppressed the inflammatory differentiation of macrophages and CD4+ T lymphocytes while reducing inflammatory factor expression (Liu et al., 2023). In chronic osteomyelitis, thorough debridement is crucial but may not completely eradicate pathogens. The presence of local microorganisms is a key cause of implant failure. By constructing a magnesium/zinc metal-organic framework coating on the titanium surface, significant inhibition of Escherichia coli and Staphylococcus aureus growth was achieved. This coating also demonstrated excellent anti-inflammatory properties, significantly improving bone formation around infected femoral implants and promoting osteogenic differentiation (Shen et al., 2019). Martin et al. found that chitosan-impregnated titanium alloy surfaces promoted cell adhesion while inhibiting biofilm formation. These surfaces enhanced osteoblast-like cell adhesion and reduced S. aureus biofilm formation by 50-75% (Villegas et al., 2022).

Implants modulate immune responses to mediate antibacterial activity and influence bone defect regeneration. Recent studies have shown that ultraviolet (UV) light significantly enhances the antibacterial activity and osteoconductive of titanium. However, the mechanisms by which UV regulates the immune system remain unclear. The combination of low-dose ozone and UV light may further enhance the antibacterial and immunomodulatory properties of titanium alloys. *In vitro* experiments demonstrated that ozone/UV-modified titanium alloys significantly enhanced macrophage phagocytosis and bactericidal activity against S. aureus. *In vivo* studies revealed that in rats with femoral defects, Micro-CT evaluation at eight weeks post-surgery showed significantly increased new bone formation, larger new bone volume, and more trabecular bone around the implant (Yang et al., 2021). Successful bone regeneration relies on effective bacterial inhibition and the induction of mesenchymal stem cell differentiation. UV/ozone-modified titanium alloys exhibit excellent antibacterial properties and superior bone immune microenvironment modulation, indicating their potential as future therapeutic strategies for bone defect treatment.

Long-term antibiotic use contributes to bacterial resistance. Biofilm-induced immune responses hinder bone and soft tissue healing, making bone infection treatment increasingly challenging. By modifying implant surface properties and using antibiotic coatings, the local antibiotic concentration around implants can be increased, effectively suppressing pathogen infections without inducing bacterial resistance (Yan et al., 2025).

## 4 3D antibacterial scaffolds

Scaffolds for bone tissue must provide structural support while also facilitating cell adhesion, proliferation, and differentiation to promote bone regeneration. However, pathogenic microorganisms can also adhere to scaffolds and develop resistance. To address this issue, antibiotic coatings and materials promoting cell proliferation and differentiation are incorporated into scaffolds (Dorati et al., 2017). Clinically, scaffolds are typically coated with combinations of two or more drugs, effectively inhibiting pathogen growth and even treating multi-pathogen infections. Additionally, dual-scaffold combination therapy strategies have been developed to simultaneously treat bone infections and bone defects while exerting anticancer effects (Vallet-Regí et al., 2020). Li et al. developed a 3D-printed MgO_2_/PLGA scaffold for postoperative osteosarcoma treatment. This scaffold induces hydrogen peroxide-mediated chemodynamic therapy, triggering tumor cell apoptosis and ferroptosis. It also polarizes macrophages toward the inflammatory M1 phenotype, activating anticancer immune responses. Moreover, Mg^2+^ released from the scaffold promotes osteogenic differentiation of bone marrow mesenchymal stem cells, aiding in bone defect healing and preventing pathogen invasion. This multifunctional scaffold, capable of anticancer, antibacterial, and bone-regenerative functions, represents a significant research focus in tumor-related biomaterials (Li et al., 2024).

Postoperative traumatic bone injuries are associated with extensive bleeding, high infection risk, and large-area bone defects that often result in bone healing failure. 3D printing technology has advanced bone tissue engineering; however, traditional 3D-printed scaffolds often possess only single functionalities, failing to meet clinical demands. Sayan et al. utilized graphitic carbon nitride as a nano-photocatalyst to enhance alginate/gelatin-based hydrogel scaffolds, achieving anti-inflammatory and bone immune modulation while exhibiting excellent hemostatic properties, which are beneficial for traumatic bone injury healing. Compared to traditional biological scaffolds, this scaffold induces macrophage polarization toward the M2 phenotype and increases IL-10 secretion, thereby promoting local healing (Dutta et al., 2025). Implant infections primarily arise due to a high local pathogen load and low antibiotic concentrations. Moreover, the increasing antibiotic resistance of S. aureus has led to higher failure rates in infection control. Qu et al. developed a 3D-printed scaffold loaded with antimicrobial peptide plasmids and antimicrobial peptides. The peptides directly killed S. aureus, while plasmid translation generated additional peptides, sustaining antibacterial activity at infection sites. This strategy alleviates immune suppression and restores protective antimicrobial immune responses, offering a novel clinical approach for S. aureus infection treatment (Qu et al., 2022). An ideal 3D-printed porous scaffold should possess mechanical strength, biodegradability, biocompatibility, and the ability to promote cell proliferation and differentiation (Dubey et al., 2023). Porous scaffolds can be soaked in various growth factors to stimulate different cellular responses *in vivo*. The properties of biomaterial scaffolds vary significantly with composition. The incorporation of cerium oxide into bioactive glass scaffolds resulted in a highly porous structure that facilitated bone ingrowth, enhancing bone strength and toughness. This scaffold also exhibited antibacterial and anti-biofilm properties against S. aureus, while its porous structure improved drug delivery capabilities (Atkinson et al., 2024). In orthopedics, infections following fractures and implant surgeries account for nearly 50% of complications, with bacterial invasion frequently leading to osteomyelitis. To avoid high-dose systemic antibiotic use, local antibiotic-loaded biological scaffolds can effectively suppress bacterial resistance formation and reduce associated complications. Maria et al. developed a biodegradable polymer-based 3D scaffold embedded with ciprofloxacin and gentamicin, along with magnesium-aluminium layered double hydroxides and α-zirconium phosphate interlayers. This scaffold simultaneously inhibited Gram-positive and Gram-negative bacterial activity while promoting bone matrix mineralization and the expression of osteogenic markers RUNX2 and OPN. The results indicate that biodegradable 3D scaffolds not only facilitate bone regeneration and bacterial inhibition but also degrade *in vivo* without requiring secondary surgical removal, reducing patient trauma (Cámara-Torres et al., 2021).

## 5 Nanocarriers

With the widespread application of 3D antibacterial scaffolds in tissue engineering and regenerative medicine, one of the challenges they face is how to further enhance the efficiency and precision of drug delivery. Recently, nanocarriers, as an emerging drug delivery system, have gradually become a key technology for addressing this issue due to their unique physicochemical properties (Din et al., 2017). Therefore, in-depth research on the application of nanocarriers in antibacterial scaffolds is of great significance for the development of next-generation high-performance biomaterials. Nanomaterials have garnered increasing attention in the medical field due to their large surface area, high biocompatibility, and broad range of applications. Nanomaterials containing various metal elements can significantly promote bone matrix mineralization, enhance antibacterial properties, and facilitate drug delivery, making them essential therapeutic carriers in bone tissue engineering (Liu et al., 2022). Various types of nano biomaterials exist, including carbon-based, metal-based, virus-based, and liposomal nanomaterials (Sharma et al., 2021). These nanomaterials are widely applied in diverse fields such as drug delivery, biosensing, chemical catalysis, and *in vivo* imaging (Fang et al., 2021). Particularly in the diagnosis and treatment of orthopedic diseases and tissue repair, nanomaterials have gained increasing recognition from clinicians and patients. Most importantly, nanomaterials exhibit low toxicity *in vivo* while improving pharmacokinetics and drug distribution, offering substantial potential for disease treatment strategies (Liu et al., 2021). To explore new methods for treating bone infections and preventing antibiotic resistance, Javier et al. developed copper-doped hollow bioactive glass nanoparticles for bone infection treatment. These nanoparticles act as nanocarriers for the broad-spectrum antibiotic danofloxacin and release copper ions. In cell and antibacterial experiments, antibiotic release was observed to slow down significantly, extending beyond one week, while effectively inhibiting the activity of Staphylococcus aureus and Escherichia coli. This effect was attributed to the synergistic action between copper ions and antibiotics, demonstrating the potential of this nano system as a localized treatment alternative for bone infections (Jiménez-Holguín et al., 2022).

Bioactive glass nanoparticles possess excellent structural properties, and modifying their ionic composition or incorporating bioactive substances can improve their biological behavior. One study investigated the biocompatibility, bone regeneration capacity, and antibacterial effects of zinc and curcumin incorporation into nanoparticles. The results demonstrated that the combination of zinc ions and curcumin significantly enhanced antibacterial properties, effectively inhibiting and degrading biofilm formation. Additionally, these components promoted the differentiation of pre-osteoblasts and mesenchymal stem cells into osteoblasts, thereby enhancing bone regeneration (Sánchez-Salcedo et al., 2023). This approach not only facilitates bone tissue healing but also addresses the issue of bacterial resistance due to antibiotic overuse. Drug delivery via biomaterials increases local drug concentration, enhances therapeutic efficacy, and reduces systemic side effects, thereby maximizing performance. Madhumathi et al. developed a dual local drug delivery system composed of calcium phosphate bioceramic nanocarriers, co-loading the antibiotic tetracycline and the anti-inflammatory drug ibuprofen to achieve both antibacterial and anti-inflammatory effects. In a rat model of cranial defect, this nanomaterial significantly promoted new bone formation at the defect site after 12 weeks compared to the control group. This combined drug delivery platform offers a comprehensive approach to managing bone infections, effectively reducing infection risk while promoting bone defect healing (Madhumathi et al., 2018). Moreover, nanomaterials play a crucial role in immune system regulation. Micellar nanocarriers loaded with mycobacterial acid were found to be phagocytosed by immune cells *in vivo*, activating immune cells. In mice, dendritic cells internalized the nanocarriers into lysosomes and subsequently activated T lymphocytes, inducing their proliferation and activation (Shang et al., 2018). Additionally, nanomaterials play a key role in enhancing neonatal innate and adaptive immunity. David et al. loaded small-molecule imidazoquinoline Toll-like receptor 8 agonists onto nanomaterials, which were internalized by dendritic cells, promoting their maturation. In animal experiments, this agonist induced T lymphocyte differentiation into CD4+ T cells, leading to an increase in immune cell populations (Dowling et al., 2017). Overall, this agonist holds significant potential as an early immune strategy against pathogens. Nanomaterials also hold a crucial position in cancer therapy. Cancer immunotherapy faces limitations due to a lack of adaptive immune response stimulation and the immunosuppressive tumor microenvironment. Guo et al. employed pH-sensitive triblock copolymers to fabricate a nanoplatform via reversible addition-fragmentation chain transfer polymerization, enabling *in situ* tumor vaccination and tumor-associated macrophage polarization. This approach mainly induced immunogenic cell death of melanoma cells through tertiary amines and thioethers on mitochondria. *In vivo* experiments demonstrated that this nanoplatform promoted dendritic cell maturation, tumor-associated macrophage polarization, and CD8+ T cell infiltration, collectively inhibiting melanoma cell proliferation and differentiation (Guo et al., 2024). Antibacterial photodynamic therapy (PDT) effectively eradicates pathogens by generating reactive oxygen species (ROS); however, excessive ROS production during treatment exacerbates oxidative stress and causes surrounding tissue damage. To address this issue, Sun et al. developed a multifunctional nanocomposite material by coating the photosensitizer chlorin e6 onto nanoceria. While chlorin e6 produced ROS to exert bactericidal effects, nanoceria scavenged excess ROS, thereby modulating the immune microenvironment by inhibiting macrophage polarization to the pro-inflammatory M1 phenotype and promoting polarization to the anti-inflammatory M2 phenotype. This effect was validated in an animal model, where inflammation and tissue regeneration at the injury site were significantly improved (Sun et al., 2021).

## 6 Hydrogels

After exploring the widespread application of nano-carriers, we further turned our attention to a material system that is closely related and has unique advantages - hydrogels. Hydrogels have become a hot field in biomedical research due to their excellent biocompatibility, adjustable mechanical properties, and good drug loading capacity. It not only serves as a carrier for nanomaterials, enhancing its functions, but also plays a key role in tissue repair and regenerative medicine. Therefore, the research on hydrogels continues the cutting-edge exploration of nanomaterials and provides a new direction for innovation in biomedical materials. Hydrogels are highly hydrophilic macromolecular materials primarily used as scaffolds for bone tissue engineering or as carriers for drug delivery in orthopedic applications. Due to their physical structure resembling bone tissue, hydrogels are well-suited for bone tissue repair. However, their large size, limited surface area, lack of microporosity, and slow degradation *in vivo* result in suboptimal performance compared to nanomaterials and titanium-based biomaterials (Zhang et al., 2023). To overcome these limitations, microgels have been developed. When injected into damaged areas, microgels form a biological protective barrier, inhibit bacterial proliferation, and serve as effective drug carriers, enhancing local drug concentration and promoting healing (Tiemeijer and Tel, 2022).

Repairing bone defects caused by infections remains a major challenge in orthopedics. Given the limited regenerative capacity of bone tissue and its slow healing process, non-union or delayed union may occur, severely impacting patients’ quality of life and mental health. Bone infections impair nerve fiber function, further hindering bone defect repair, as certain growth factors involved in osteogenesis are neuronally regulated. Jing et al. incorporated magnesium-modified black phosphorus into gelatin methacrylate to fabricate a photosensitive conductive hydrogel. Black phosphorus exhibited potent antibacterial activity by modulating the immune microenvironment of the infection site and reducing virulence factor secretion that damages bone and surrounding tissues. Simultaneously, magnesium ions promoted Schwann cell migration and secretion, facilitating neurite outgrowth and subsequent bone regeneration. In a rat model of infected cranial defect, this hydrogel demonstrated effective antibacterial properties while promoting nerve fiber and bone tissue regeneration (Jing et al., 2023). Various biomaterials are available in the market for clinical applications, yet the risk of implant-associated infections is increasing. Dysregulated host immune responses can even lead to failed bone healing. Therefore, developing novel bone biomaterials with antimicrobial and immunoregulatory properties is critical. One study encapsulated W9 peptides in a hydrogel containing LL37 peptides and poly (lactic-co-glycolic acid) microspheres. *In vitro* experiments demonstrated sustained drug release, excellent antibacterial properties, and the ability to recruit bone marrow stem cells. The hydrogel initially induced macrophage polarization to the pro-inflammatory M1 phenotype, facilitating early infection clearance. Subsequently, W9 peptides promoted macrophage polarization to the anti-inflammatory M2 phenotype, suppressing pro-inflammatory gene expression and supporting bone tissue healing, as observed in a rat cranial defect model (Ma et al., 2022; Chen et al., 2023).

Long-term diabetes leads to necrosis of blood vessels and nerves in the lower limbs, particularly in the feet, ultimately resulting in foot ulcers accompanied by persistent bacterial infections. Prolonged immune inflammation and delayed healing of soft tissues and bones pose significant challenges in clinical management. Ge et al. integrated silver nanoclusters with exosomes derived from M2 macrophages to develop a DNA-based multifunctional hydrogel with antibacterial, anti-inflammatory, and osteogenic properties. This material effectively prolonged the retention time of exosomes *in vivo*, accelerated bone defect healing, and significantly increased the number of trabeculae, demonstrating that the composite hydrogel can effectively regulate macrophage polarization toward the anti-inflammatory M2 phenotype and enhance the expression of osteogenic factors (Peng et al., 2024). Diabetic foot ulcers can lead to irreversible damage, such as nerve and vascular degeneration. Even after amputation, skin healing remains a challenge, particularly in bone tissue repair and regeneration. Wu et al. developed an injectable and photopolymerizable hydrogel therapy platform based on alginate-functionalized Ti3C2 nanosheets for mediating bone regeneration, coordinating immune responses, and providing antibacterial effects. The optimized hydrogel suppressed reactive oxygen species (ROS)-induced inflammatory responses by modulating the balance of M1/M2 macrophages. Additionally, the thermally responsive hydrogel effectively inhibited local immune responses to promote bone regeneration. Notably, even in the absence of exogenous cytokines and growth factors, the hydrogel maintained its regenerative capability, offering a promising therapeutic strategy for bone tissue engineering and regenerative medicine (Wu et al., 2023).

## 7 Discussion

In the field of tissue engineering, an increasing number of biomaterials are being developed, as traditional medical biomaterials can no longer fully meet clinical and patient needs. One of the major challenges faced by clinicians and researchers is identifying novel alternative materials to combat clinical tissue infections. Currently, biomaterials primarily focus on antibacterial and regenerative therapies. In terms of immune modulation, they mainly mediate the immune microenvironment by regulating macrophage polarization and stimulating T lymphocyte differentiation, thereby inhibiting the proliferation of pathogenic microorganisms and promoting the healing and repair of bone and surrounding soft tissues. Immune modulation strategies have demonstrated broad application prospects in the treatment of bone infections. Recently, studies have shown that the immune system plays a key role in the defines mechanisms against bone infections. By modulating the host’s immune response, immune modulation strategies hold promises as an important means to address the issues of antibiotic resistance and biofilm-associated infections (Seebach and Kubatzky, 2019). For instance, modulating the activity of T cells and myeloid-derived suppressor cells (MDSCs) has been shown to improve the therapeutic outcomes of chronic implant-associated bone infections. Additionally, the successful application of immune checkpoint inhibitors in cancer treatment has provided new insights for the treatment of bone infections (Lesokhin et al., 2013a). However, immune modulation strategies also pose potential risks. First, immune modulating drugs may trigger systemic immune responses, leading to exacerbated inflammatory reactions in non-infected sites. Second, immune modulation strategies may interfere with normal bone metabolism processes; for example, overactivation of immune cells may lead to increased bone resorption. Furthermore, long-term use of immune-modulating drugs may result in immune tolerance or immune suppression, thereby increasing the risk of patients contracting other pathogens (Su et al., 2022; Vantucci et al., 2022). Immune modulation strategies also involve a range of ethical issues. For instance, the use of immune modulating drugs may have long-term impacts on the patient’s immune system, the full extent of which may be difficult to predict. Moreover, immune modulation strategies may increase the patient’s susceptibility to other diseases, which requires careful weighing during the treatment process. In clinical applications, it is essential to ensure that patients are fully informed of the risks and potential benefits of the treatment, and that treatment is conducted based on informed consent (Seebach and Kubatzky, 2019).

It is worth noting that gene-editing technologies, such as CRISPR-Cas9, provide powerful tools for the precise regulation of immune cell functions. Through gene editing, immune cells (such as macrophages and T cells) can be engineered to exert stronger antibacterial and anti-inflammatory effects at the site of bone infection. For example, studies have shown that enhancing the M2 phenotype of macrophages via gene editing can effectively reduce inflammatory responses and promote bone regeneration (Su et al., 2022; Deneault, 2024). Additionally, gene editing can also be used to develop novel immunomodulatory drugs that can specifically target the site of infection, thereby reducing systemic side effects. The emerging artificial intelligence (AI) technologies can predict patients’ responses to immunomodulatory treatments by analysing vast amounts of clinical data and biomarkers, thereby enabling personalized treatment plans. AI algorithms are capable of identifying which patients are more likely to benefit from specific immunomodulatory strategies while also forecasting potential adverse reactions. Although it has not yet been put into clinical use, it can be an important direction for future consideration.

Future research should focus on how these emerging technologies can be combined with existing immunomodulatory strategies to improve the safety and effectiveness of treatments. For example, developing intelligent biological materials based on AI that can dynamically regulate immune responses based on the microenvironment of infected sites.

Traditional biomaterials have attempted to achieve antibacterial properties by modifying their physical characteristics, such as incorporating antibiotic coatings or increasing porosity to facilitate immune cell infiltration (Pelgrift and Friedman, 2013). Compared to conventional biomaterials, newly emerging biomaterials in recent years, such as nanocarriers and hydrogels, have demonstrated multifunctionality by carrying different drugs or bioactive materials *in vivo* (Lesokhin et al., 2013b; Tang and Zheng, 2018). These materials can inhibit cancer cell proliferation and differentiation, promote the repair of bone defects, and modulate the immune microenvironment at infection sites. With advancements in science and technology, biomaterials have become increasingly diverse. By optimizing their properties, biomaterials can maintain excellent biocompatibility, ensuring that the host does not trigger rejection responses while simultaneously addressing infection and healing challenges. Scaffolds, in particular, offer significant advantages in preventing infections and treating skin and bone tissue defects (Gutierrez et al., 2022). Traditional biomaterials have been well-validated and widely supported in clinical applications. However, a major limitation of novel biomaterials is their lack of extensive clinical application, as their use remains largely restricted to *in vitro* cell and bacterial experiments or certain animal models, with insufficient clinical data to substantiate their efficacy. Nevertheless, the progress in tissue engineering is evident. In a certain sense, compared to traditional therapies, biomaterials offer a more personalized and adaptable approach to meeting the clinical needs of each patient.

With the increasing severity of antibiotic resistance, the development of new antibiotics has become a crucial direction for the treatment of bone infections. For example, some novel antibiotics such as daptomycin and oritavancin have shown good efficacy in clinical trials for treating complex bone and joint infections (Olmos and Gonzalez-Benito, 2021; Wang et al., 2024). These antibiotics not only possess strong antibacterial activity but can also effectively penetrate biofilms, reducing the emergence of resistant strains. Additionally, rifampin has achieved significant results in the treatment of Staphylococcus aureus-related bone infections, with combination therapy strategies significantly reducing the recurrence rate. In terms of new immunotherapy, the successful application of immune checkpoint inhibitors in cancer treatment has also provided new directions for the treatment of bone infections. Studies have shown that modulating the activity of T cells and myeloid-derived suppressor cells (MDSCs) can improve the therapeutic outcomes of chronic implant-associated bone infections (Hu et al., 2024).
